# Nature and mechanisms of hepatocyte apoptosis induced by d-galactosamine/lipopolysaccharide challenge in mice

**DOI:** 10.3892/ijmm.2014.1730

**Published:** 2014-04-07

**Authors:** YI-HANG WU, SHAO-QING HU, JUN LIU, HONG-CUI CAO, WEI XU, YONG-JUN LI, LAN-JUAN LI

**Affiliations:** 1 Zhejiang Provincial Key Laboratory of Biometrology and Inspection and Quarantine, Department of Pharmacy, College of Life Sciences, China Jiliang University, Hangzhou, Zhejiang 310018, P.R. China; 2 State Key Laboratory for Diagnosis and Treatment of Infectious Diseases, The First Affiliated Hospital, College of Medicine, Zhejiang University, Hangzhou, Zhejiang 310003, P.R. China

**Keywords:** d-galactosamine, lipopolysaccharide, hepatocyte apoptosis, toxicologic pathology, mechanism of apoptosis, mouse

## Abstract

Apoptosis plays a role in the normal development of liver. However, overactivation thereof may lead to hepatocellular damage. The aim of this study was to assess d-galactosamine (d-GalN)/lipopolysaccharide (LPS)-induced hepatocyte apoptotic changes in mice and clarify the mechanisms involved in this process. DNA ladder detection was employed to determine the induction condition of hepatic apoptosis. An initial test indicated that typical hepatocyte apoptosis was observed at 6–10 h after the intraperitoneal injection of d-GalN (700 mg/kg) and LPS (10 μg/kg). Subsequently, we evaluated hepatocyte apoptosis at 8 h after administering d-GalN/LPS by histopathological analysis, terminal deoxynucleotidyl transferase-mediated dUTP nick end-labeling (TUNEL) detection, flow cytometry and electron microscopy analysis. To clarify the apoptosis-related gene expression, the expression levels of tumor necrosis factor-α (TNF-α), transforming growth factor-β1 (TGF-β1), caspase-3, and Fas/Fas ligand (FasL) were determined by serum enzyme immunoassay, immunohistochemistry and western blot analysis. Strong apoptotic positive signals following d-GalN/LPS injection were observed from the results of the serum analysis, histopathological and immunohistochemical analyses, DNA ladder detection, TUNEL detection, flow cytometry and electron microscopy analysis. Additionally, apoptotic hepatocytes were mainly at the late stage of cell apoptosis. The expression of TNF-α, TGF-β1, caspase-3 and Fas/FasL was significantly increased. In conclusion, this study evaluated the d-GalN/LPS-induced hepatocyte apoptotic changes and clarified the apoptosis-related gene expression in mice. The hepatocyte apoptosis induced by d-GalN/LPS may be mainly regulated by the death receptor pathway. TGF-β signaling pathway may also play a vital role in this process of hepatocyte apoptosis.

## Introduction

Apoptosis is essential in many aspects of normal development and is required for maintaining homeostasis (1). In liver, apoptosis is a physiological process involved in the clearance of injured cells and in homeostatic control (2). Physiologically, apoptosis is virtually undetectable in the liver, with only 1–5 apoptotic cells/10,000 cells detected (3). In the context of liver disease, overactivation of the apoptotic process may lead to hepatocellular damage, while the inhibition of apoptosis may promote cell proliferation and transformation. Fulminant hepatic failure, induced by drugs, toxins or viral hepatitis, is characterized by severe hepatocellular dysfunction with a massive death of hepatocytes, in which apoptosis may play a role in addition to necrosis (4). d-galactosamine (d-GalN) is a specific hepatotoxic agent metabolized exclusively in hepatocytes, which reduces the intracellular pool of uracil nucleotides, thus inhibiting the synthesis of RNA and proteins (5). When administered in combination with a low dose of lipopolysaccharide (LPS), d-GalN highly sensitizes animals to develop lethal liver injury, showing biochemical and metabolic changes akin to fulminant hepatic failure (6). d-GalN and the LPS-induced liver failure model takes advantage of the ability of a transcriptional inhibitor d-GalN to potentiate the toxic effects of LPS, producing typical hepatic necrosis and apoptosis followed by fulminant hepatitis (7). Although typical hepatic apoptosis has been shown to emerge in the d-GalN/LPS-induced liver damage model, the hepatocyte apoptotic changes induced by d-GalN/LPS are not evaluated systematically and its mechanisms are poorly understood.

The role of apoptosis in various liver diseases and the mechanisms by which apoptosis occurs in the liver may provide insight into these diseases and suggest possible treatments (8). The aim of the present study was to clarify d-GalN/LPS-induced hepatocyte apoptotic changes and the related gene expression using serum analysis, histopathological and immunohistochemical analyses, DNA ladder detection, terminal deoxynucleotidyl transferase-mediated dUTP nick end-labeling (TUNEL) detection, western blot analysis, flow cytometry and electron microscopy analysis.

## Materials and methods

### Reagents

LPS *Escherichia coli* 005:B5 and d-GalN were purchased from Sigma Chemical Co. (St. Louis, MO, USA). The alanine aminotransferase (ALT) diagnostic kit was provided by Nanjing Jiancheng Bioengineering Institute (Nanjing, China). The apoptosis DNA ladder detection kit and apoptotic cell Hoechst 33342/propidium iodide (PI) detection kit were obtained from Nanjing KeyGen Biotech Co., Ltd. (Nanjing, China). Caspase-3 primary antibody, Fas/Fas ligand (FasL) primary antibodies, mouse tumor necrosis factor-α (TNF-α) enzyme immunoassay kit and mouse transforming growth factor-β1 (TGF-β) enzyme immunoassay kit were purchased from Boster Biological Technology, Ltd. (Wuhan, China). The mouse SP-9002 immunohistochemical detection kit was provided by Beijing Zhongshan-Golden Bridge Biological Technology Co., Ltd. (Beijing, China). The goat anti-rabbit IgG-horseradish peroxidase (HRP) and glyceraldehyde-3-phosphate dehydrogenase (GAPDH) were obtained from Santa Cruz Biotechnology, Inc. (Santa Cruz, CA, USA). ECL substrate kit for western blot analysis was purchased from Pierce Biotechnology Inc. (Rockford, IL, USA). *In situ* cell death detection kit, POD was provided by Roche Diagnostics GmbH (Mannheim, Germany). Tris-HCl, Coomassie brilliant blue R250 was provided by Amresco Inc. (Solon, OH, USA). All other reagents were of the highest commercial grade available.

### Animals

Male ICR mice weighing 20–25 g were provided by the Experimental Animal Center of Zhejiang Province. The mice were kept in a room maintained at 22±2°C and at relative humidity of 40–70%. The animal experimental protocol was approved by the Animal Ethics Committee of Zhejiang University, in accordance with the Guiding Principles in the Use of Animals in Toxicology, adopted by the Society of Toxicology (USA) in July 1989 and revised in March 1999.

### Induction of hepatocyte apoptosis by d-GalN/LPS in mice

**Figure 1:**
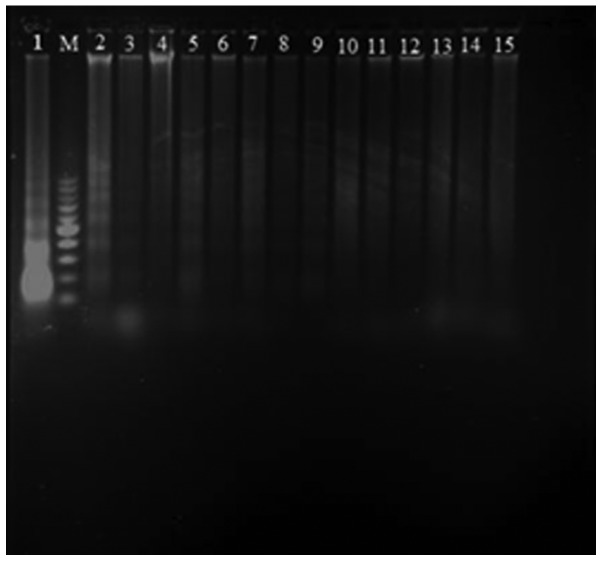
Analysis of DNA ladder from the liver tissues at 6–24 h after the d-galactosamine (d-GalN)/lipopolysaccharide (LPS) injection. Lane M, marker (molecular weight standard); lanes 1–3, isolated liver cells at 6 h; lanes 4–6, isolated liver cells at 8 h; lanes 7–9, isolated liver cells at 10 h; lanes 10–12, isolated liver cells at 12 h; and lanes 13–15, isolated liver cells at 24 h after the d-GalN/LPS treatment.

To induce hepatocyte apoptosis by d-GalN/LPS, DNA ladder detection was employed as the evaluation method. Analysis of DNA fragmentation indicated that the typical hepatocyte apoptosis was observed at 6–10 h after the intraperitoneal injection of d-GalN (700 mg/kg) and LPS (10 μg/kg) (Fig. 1). Therefore, the liver apoptosis model was established as follows: the animals were divided into the vehicle control group (n=7) and the model group (n=7). The mice in model group were injected intraperitoneally with d-GalN (700 mg/kg) and LPS (10 μg/kg) dissolved in normal saline. The vehicle control groups received the same volume of normal saline. Eight hours after the last administration, the mice were slightly anaesthetized with ether and blood samples were obtained from the eyepit. The animals were sacrificed by cervical dislocation and liver samples were collected for further examination.

### Determination of serum ALT, TNF-α and TGF-β1 levels

The serum was separated for the measurement of ALT, TNF-α and TGF-β1. According to the manufacturer’s instructions, the serum ALT activity was determined using the ALT detection kits; the serum TNF-α and TGF-β1 levels were quantified by the murine TNF-α and TGF-β1 enzyme immunoassay kits, respectively.

### Liver histology and immunohistochemistry

For histopathological analysis, the liver specimens were fixed in 10% neutral-buffered formalin, embedded in paraffin, cut into 5-μm sections, and stained with hematoxylin and eosin (H&E). For the immunohistochemical analysis of caspase-3, Fas and FasL, the staining steps of the paraffin sections following dewaxing and hydration were as follows: the sections were incubated in 0.3% H_2_O_2_-methanol solution for 10 min and then washed with phosphate-buffered saline (PBS) three times. To block endogenous peroxidase activity, 50 μl of peroxidase blocking solution was added to each section and incubated for 10 min at room temperature. Each section was then incubated with 50 μl of non-immune goat serum for 10 min at room temperature, rinsed with PBS and incubated with 50 μl of primary antibody (rat monoclonal IgG for caspase-3 or rabbit polyclonal IgG for FAS and FasL) at 4°C overnight. The sections were then incubated with 50 μl of biotin-labeled secondary antibody (Bio-IgG) at room temperature for 10 min. After washing three times with Tris-buffered saline (TBS), each section was overlaid with 50 μl of SP-9002 (peroxidase-conjugated goat anti-mouse IgG) solution at room temperature for 10 min. The reaction was developed with freshly prepared 3,3′-diaminobenzidine (DAB), and observed under a Leica DM4000 microscope (Leica Microsystems, Wetzlar, Germany) for 3–10 min, with brown or red indicating positive. The slides were then counterstained with hematoxylin. The pathological and immunohistochemical changes were evaluated and photographed under a Leica DM4000 microscope.

### Analysis of DNA fragmentation of apoptotic cells

The collected liver specimens were washed and then homogenized in cold PBS. The homogenate was transfered to a 1.5 ml microcentrifuge (Eppendorf) tube. The DNA of liver tissue was extracted using the apoptosis DNA ladder detection kit as per the manufacturer’s instructions. The DNA fragmentation was assayed by electrophoresis on a 1.5% agarose gel containing 0.5 μg/ml ethidium bromide and its pattern was examined on the images obtained under ultraviolet illumination.

### TUNEL detection of apoptotic cells

The TUNEL assay was used to quantify liver apoptosis and was performed using the *in situ* cell death detection POD kit according to the manufacturer’s instructions. Briefly, paraffin-embedded tissue sections were dewaxed and rehydrated according to the standard protocols (e.g., by heating at 60°C followed by washing in xylene and rehydration through a graded series of ethanol and double distilled water). The sections were then incubated with proteinase K (20 μm/ml in 10 mM Tris-HCl, pH 7.4) for 30 min at 37°C. Slides were rinsed with PBS and incubated with 3% H_2_O_2_ in methanol for 30 min at room temperature to block endogenous POD activity, followed by PBS washing and incubation in 0.1% Triton X-100 in 0.1% sodium citrate for 2 min at 4°C. After washing in PBS, the sections were incubated with the TUNEL reaction mixture containing terminal deoxynucleotidyl transferase in a moist chamber at 37°C for 60 min in the dark. This was followed by washing with PBS and incubation with converter-POD (anti-fluorescein-HRP) in a humidified chamber at 37°C for 30 min. After washing with PBS, the sections were incubated with DAB substrate for 10 min at room temperature, rinsed again with PBS, mounted under glass coverslip and analyzed under a light microscope. The negative control was obtained by replacing the TdT solution with distilled water.

### Western blot analysis for Fas, FasL and caspase-3 expression

Total proteins from the collected liver samples were prepared according to the method described in the protein extract kit. Protein concentrations were determined by Coomassie blue dye-binding assay. Protein extracts were fractionated on 12% polyacrylamide-sodium dodecyl sulfate gel and then transferred to a polyvinylidene fluoride membrane. The membrane was blocked with 5% (w/v) fat-free milk in TBS containing 0.05% Tween-20, followed by incubation with rat primary anti-FAS (1:150), anti-FasL (1:150) and anti-caspase-3 (1:150) polyclonal antibody at room temperature for 2 h, respectively. After washing the membrane with TBS, the membrane was treated with HRP-conjugated goat anti-rat secondary antibody IgG-HRP (1:3,000) for 1 h at room temperature via agitation. The enhanced HRP-DAB substrate solution was added to the membrane and incubated for 10 min. Bands were visualized by chemiluminescence and exposed to X-ray. The GAPDH antibody was used as an internal control. The relative optical density (ROD, ratio to GAPDH) of each blot band was quantified by BandScan 4.5 image software.

### Flow cytometric analysis of apoptotic cells

Apoptotic cells in the liver tissue were quantified by flow cytometry. Briefly, the collected liver specimens were homogenized in cold PBS. The homogenate was filtered through nylon net, centrifuged and washed with PBS twice. The collected cells were resuspended in RPMI-1640 medium and adjusted to 5×10^5^ cells/ml. According to the manufacturer’s instructions, the cell suspension was double-stained with Heochst 33342 at 37°C for 10 min and PI for 10 min at 37°C in the dark, respectively. Apoptotic analysis was immediately performed on a BD FACSAria flow cytometer (Becton-Dickinson and Co., Franklin Lakes, NJ, USA). The population was separated into three groups: live cells showing only a low level of fluorescence; apoptotic cells showing a higher level of blue fluorescence, and dead cells showing low-blue and high-red fluorescence.

### Electron microscopy analysis of apoptotic cells

For electron microscopy examination, the collected liver tissues were cut into ~1 mm^3^ and fixed in 2.5% glutaraldehyde for 2 h. After rinsing with PBS, the tissues were fixed in 1.5% osmium tetroxide, dehydrated through a graded alcohol, embedded in epon 812, ultrathin-sectioned, and stained with uranyl acetate and lead citrate. Labeled ultrathin sections were observed and images were captured under a JEM-1200EX transmission electron microscope (Jeol Ltd., Tokyo, Japan).

### Statistical analysis

Experimental data were expressed as mean ± standard deviations (SD) and subjected to a one-way analysis of variance (ANOVA) and the Student’s t-test. P<0.05 was considered to indicate statistical significance.

## Results

### Serum ALT, TNF-α and TGF-β1 levels

ALT is a vital parameter for liver necrosis, whereas TNF-α and TGF-β1 are the critical mediators of liver apoptosis. We hypothesized that the levels of three mediators would increase in mice after the d-GalN/LPS challenge. The results showed that the serum ALT, TNF-α and TGF-β1 levels of the d-GalN/LPS-treated mice were significantly elevated with respect to the vehicle control (Table I).

### Liver histopathology

**Figure 2:**
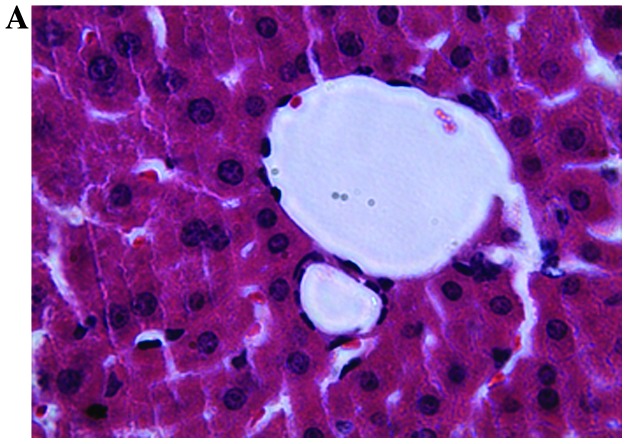

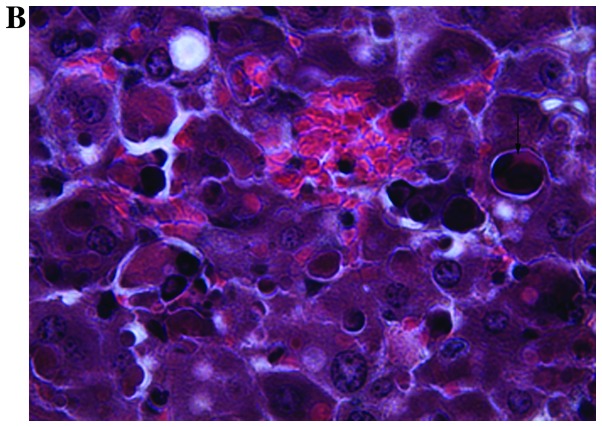
Histopathological analysis of d-galactosamine (GalN)/lipopolysaccharide (LPS)-induced hepatocyte apoptosis model in mice [hematoxylin and eosin (H&E) ×400]. (A) Normal central vein and hepatocyte structure in untreated control mouse; (B) d-GalN/LPS-treated mouse showing apoptotic liver cells (arrowhead) and hepatic necrosis with leukocytes infiltration etc.

Histopathological analysis showed that hepatic lobular architecture was clear and intact without any abnomalities in the liver section of the control group. At 8 h after d-GalN/LPS injection, large apoptotic liver cells and sections of hepatic necrosis with leukocyte infiltration were found (Fig. 2). Strong apoptotic positive signals including chromatin condensation and margination, disruption of nuclear membrane, and fragmentation of chromatin and the formation of apoptotic bodies was observed.

### Liver immunohistochemistry

**Figure 3:**
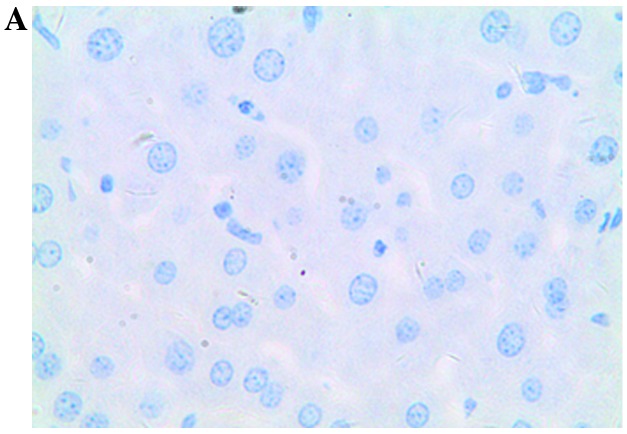

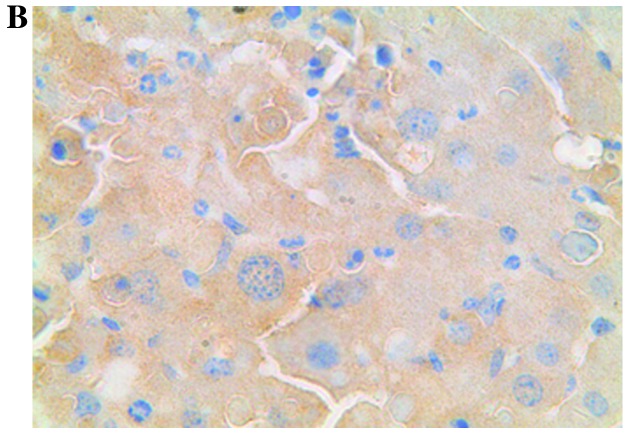
Immunohistochemical analysis of caspase-3 expression of the apoptotic hepatocyte (magnification, ×400). (A) Untreated control mouse; (B) d-galactosamine (d-GalN)/lipopolysaccharide (LPS)-treated mouse showing the positive expression of caspase-3 (brown stain).

**Figure 4:**
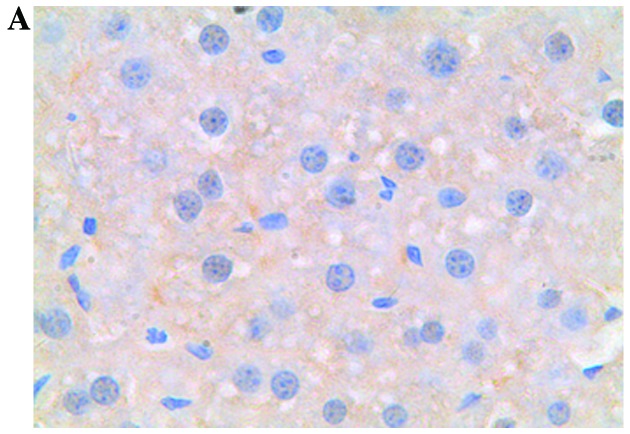

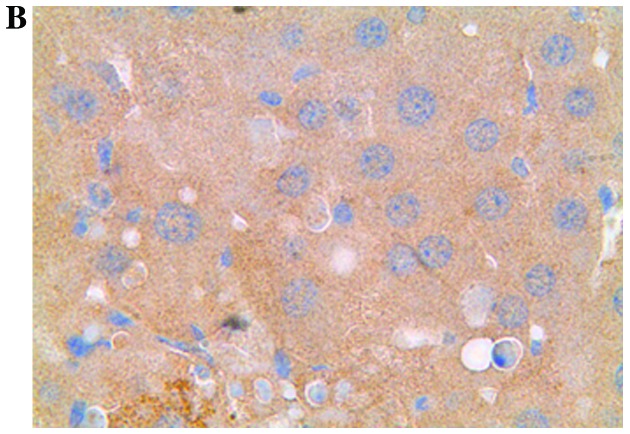
Immunohistochemical analysis of Fas expression of the apoptotic hepatocyte (magnification, ×400). (A) Untreated control mouse; (B) d-GalN/lipopolysaccharide (LPS)-treated mouse showing the positive expression of Fas receptor (brown stain).

**Figure 5:**
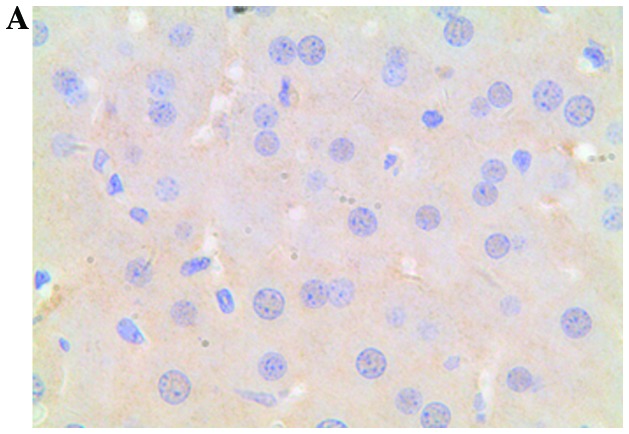

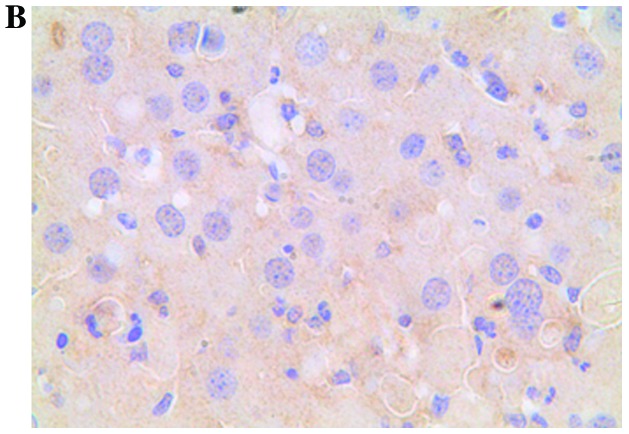
Immunohistochemical analysis of Fas ligand (FasL) expression of the apoptotic hepatocyte (magnification, ×400). (A) Untreated control mouse; (B) d-galactosamine (d-GalN)/lipopolysaccharide (LPS)-treated mouse showing the positive expression of FasL (brown stain).

Caspase-3 plays a central role in the execution phase of cell apoptosis. The Fas/FasL system provides a major apoptotic mechanism for many cell types, including liver cells. We examined the expression of caspase-3, Fas and FasL of the isolated liver at 8 h after d-GalN/LPS treatment. Immunohistochemical analysis revealed that d-GalN/LPS treatment markedly increased hepatic expression of caspase-3, as well as the pro-apoptotic receptors Fas and FasL in mice (Figs. 3–5).

### Analysis of DNA fragmentation

**Figure 6:**
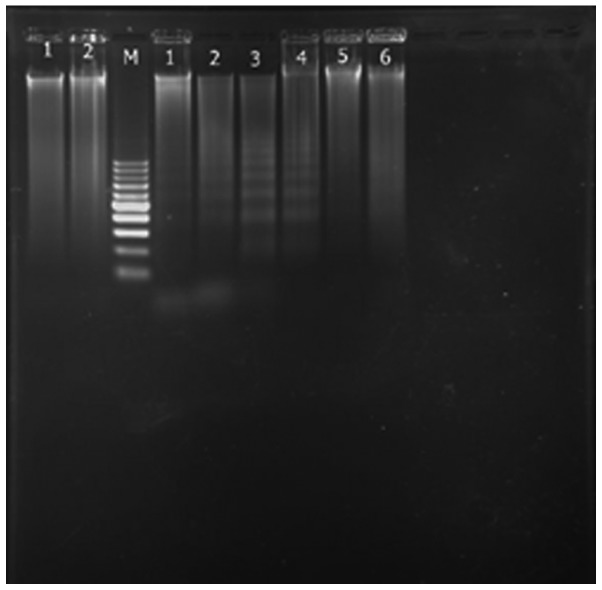
Analysis of DNA ladder from the liver tissues at 8 h after the d-galactosamine (d-GalN)/lipopolysaccharide (LPS) injection. Lane M, marker (molecular weight standard); lanes 1 and 2 to the left of lane M, normal liver cells; lanes 1–4 to the right of lane M, isolated liver cells at 8 h after d-GalN/LPS treatment.

Genomic DNA fragmentation was assayed to confirm the occurrence of hepatocyte apoptosis. DNA fragmentation was observed in murine liver after d-GalN/LPS treatment, while no DNA fragmentation was found in the untreated control untreated (Fig. 6).

### TUNEL detection of apoptotic cells

**Figure 7:**
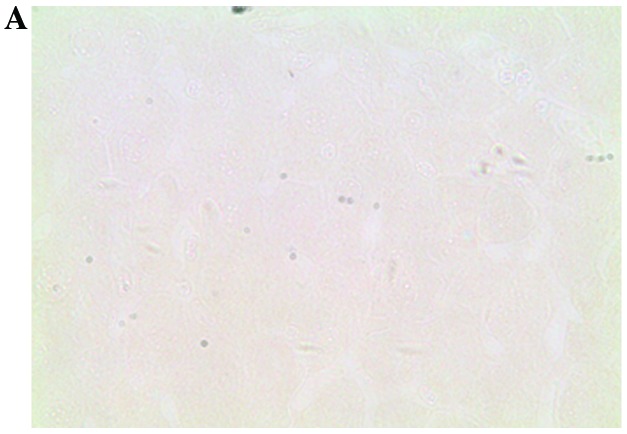

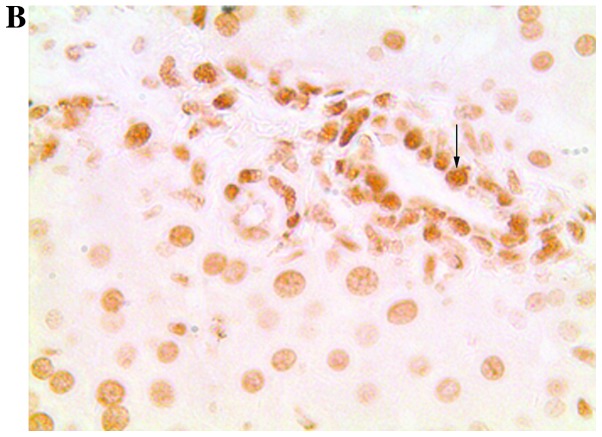
TUNEL detection of apoptotic hepatocytes (magnification, ×400). (A) Untreated control mouse; (B) d-galactosamine (d-GalN)/lipopolysaccharide (LPS)-treated mouse showing apoptotic liver cells (arrowhead).

Apoptotic cells were detected by TUNEL staining. A large number of TUNEL-positive hepatocytes were observed in liver tissues from mice following d-GalN/LPS treatment. However, no TUNEL-positive hepatocytes were observed in the livers from the untreated control mice (Fig. 7).

### Western blot analysis for Fas, FasL and caspase-3 expression

**Figure 8:**
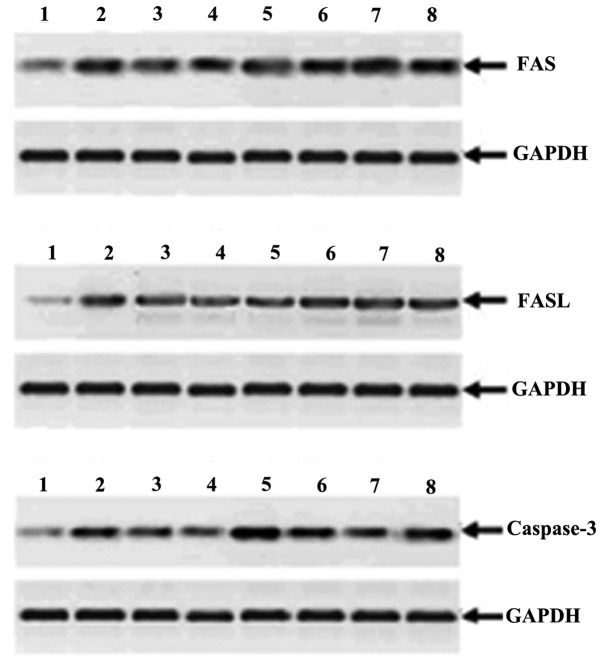
Western blot analysis of Fas, Fas ligand (FasL) and caspase-3 expression in liver tissues. The GAPDH antibody was used as internal control. Sample 1 from the untreated control mice; Samples 2–8 from the mice at 8 h after the d-galactosamine (d-GalN)/lipopolysaccharide (LPS) injection.

The expression levels of Fas, FasL and caspase-3 in liver tissues from the mouse after d-GalN/LPS treatment were quantified by western blotting. The GAPDH antibody was used as an equal loading internal control. As shown in Fig. 8 and Table II, compared with the untreated control mice, the expression levels of caspase-3, Fas and FasL were markedly enhanced after the d-GalN/LPS treatment.

### Flow cytometric analysis of apoptotic cells

**Figure 9:**
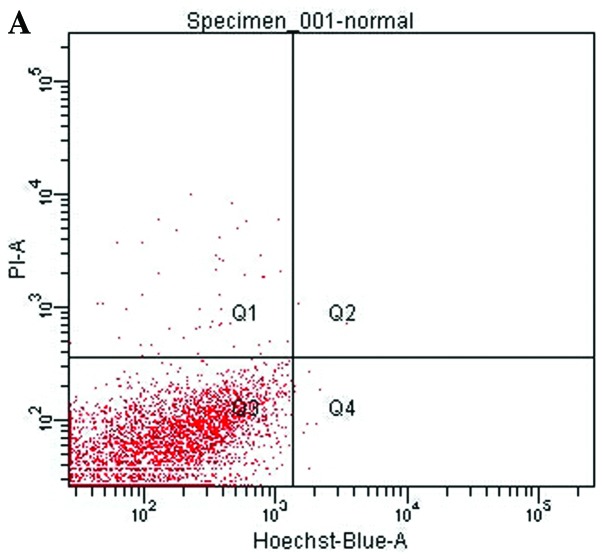

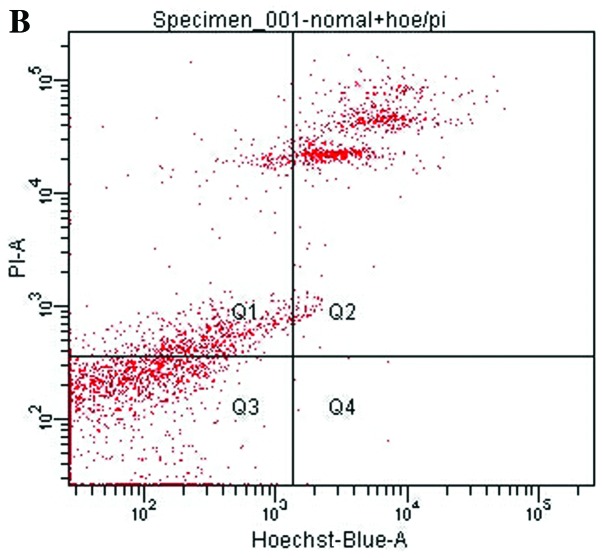
Flow cytometric analysis of apoptotic liver cells. (A) Untreated control mouse; (B) d-galactosamine (d-GalN)/lipopolysaccharide (LPS)-treated mouse. Live cells show only a low level of fluorescence; apoptotic cells show a higher level of blue fluorescence, and dead cells show low-blue and high-red fluorescence. Q1 district showing injury cells; Q2 district showing necrotic cells and apoptotic cells at late stage; Q3 district showing normal live cells; Q4 district showing apoptotic cells at early stage.

The hepatocytes were isolated from the murine liver following administration of d-GalN/LPS and staining with Hoechst 33342. Hoechst 33342, a type of blue-fluorescence dye, stains the condensed chromatin in apoptotic cells more brightly than normal chromatin. PI a red-fluorescence dye, is only permeant to dead cells. The staining pattern resulting from the simultaneous use of these dyes makes it possible to distinguish normal, apoptotic, and dead cell populations by flow cytometry. The result showed that the injury, necrotic and apoptotic cells were observed following d-GalN/LPS treatment and that apoptotic cells were mainly at the late stage of cell apoptosis (Fig. 9).

### Electron microscopy analysis of apoptotic cells

**Figure 10:**
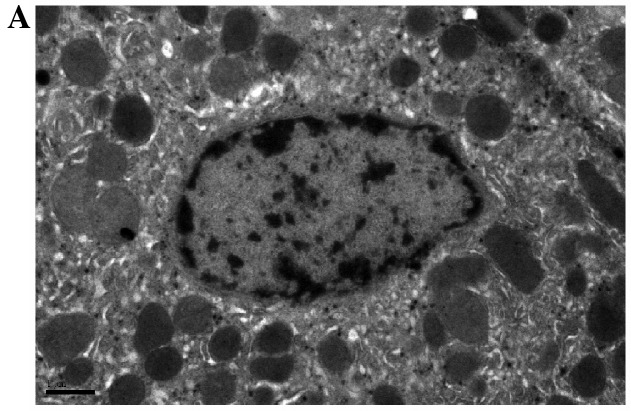

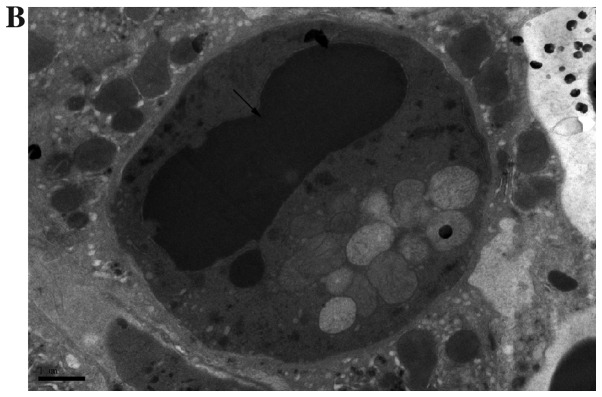
Transmission electron microscopy analysis of apoptotic liver cells (magnification, ×15,000). (A) Untreated control mouse showing normal hepatocyte structure; (B) d-galactosamine (d-GalN)/lipopolysaccharide (LPS)-treated mouse showing chromatin condensation in apoptotic cells to form crescent moon apoptotic body (arrowhead) located near the nucleus membrane.

To analyze the typical morphological signs of apoptosis, the ultrathin sections from the mouse liver tissues after administering D-GalN/LPS were observed under electron microscope. The chromatin of apoptitic liver cell appeared condensation, approaching to nuclear membrane, gathering to the edge and forming typical apoptosis body (Fig. 10).

## Discussion

Liver disease is often associated with enhanced hepatocyte apoptosis, which is the case in viral and autoimmune hepatitis, cholestatic diseases, and metabolic disorders. Use and abuse of certain drugs, especially alcohol, chemotherapeutic agents, and acetaminophen, have been associated with increased apoptosis and liver damage (8). The availability of animal models relevant to apoptosis may facilitate the identification of potential therapies. d-GalN is a typical hepatotoxin and often used in pharmacodynamics research to induce hepatic injury. This model of liver damage most closely resembles the changes observed during human hepatitis, and thus provides a useful system for screening and investigating drugs that can be used in the treatment of disease (5). LPS, the major structural component of the outer membrance of gram-negative bacteria, causes liver injury at high doses but a modest, non-injurious inflammation at low doses in several animal models (9–11). In addition, LPS can induce lethal liver failure when simultaneously administered with d-GalN (7). In this investigation, we reported the nature of d-GalN/LPS-induced hepatocyte apoptotic changes and the related gene expression in mice.

DNA ladder detection indicated that hepatocyte apoptosis occurred at 6–10 h after the intraperitoneal injection of d-GalN (700 mg/kg) and LPS (10 μg/kg). Based on the initial test, we demonstrated the occurrence of the hepatocyte apoptosis at 8 h after administering d-GalN/LPS by histopathological analysis, TUNEL detection, flow cytometry and electron microscopy analysis. Histopathological analysis showed that strong apoptotic positive signals were observed including chromatin condensation and margination, disruption of nuclear membrane, and fragmentation of chromatin and the formation of apoptotic bodies. TUNEL detection indicated that a large number of TUNEL-positive hepatocytes were observed in liver tissues. Flow cytometric analysis showed that the apoptotic liver cells were mainly at the late stage of cell apoptosis, suggesting that DNA fragmentation may occur at late stage of cell apoptosis. Electron microscopy analysis indicated the typical morphological signs of apoptosis especially the formation of apoptotic bodies. These results suggest that typical hepatocyte apoptosis was definitely induced by d-GalN/LPS in mice.

Overactivation involving mediators such as Fas, TNF-α and TGF-β, can lead to significant acute injuries, such as fulminant hepatic failure or even chronic sustained hepatocellular damage, as occurs with toxic liver injury, viral hepatitis, alcoholic and non-alcoholic liver disease (12). To investigate apoptosis-related gene expression changes, we examined the expression of TNF-α, TGF-β1, Fas, FasL and caspase-3 by serological measurement, immunohistochemical analysis and western blot analysis. The serological detection showed that the ALT, TNF-α and TGF-β1 levels of the d-GalN/LPS-treated mice were significantly enhanced, suggesting that apoptosis and necrosis occurred together following d-GalN/LPS challenge. Immunohistochemical analysis indicated that d-GalN/LPS treatment markedly increased the expression of caspase-3, as well as the pro-apoptotic receptors Fas and FasL in mice. Western blot analysis showed the relative expression of Fas, FasL, and caspase-3 proteins, which were markedly elevated after d-GalN/LPS treatment. The above results suggest that the apoptosis-related gene expressions (TNF-α, TGF-β1, Fas/FasL and caspase-3) were significantly increased following d-GalN/LPS treatment.

Apoptosis plays an important role in liver pathogenesis, and disarrangement of death receptor pathways has been identified as a major contributor to the initiation and aggravation of acute and chronic liver injury (12). Apoptotic signalling within the cells is transduced mainly via two molecular pathways: the extrinsic or death receptor pathway and the intrinsic or mitochondrial pathway. Apoptotic events in hepatocytes can be regulated by different stimuli that bind to death receptors in the cell membranes, such as FasL, TNF or TNF-related apoptosis-induced ligand, which activate the extrinsic pathway. Binding of FasL or TNF-β to their corresponding death receptors induce the recruitment of procaspase 8–10 to form the death-inducing signaling complex, leading to cell death (13). Other factors, particularly TGF-β, do not bind the death receptors, but its intracellular signals couple to the apoptotic machinery through activation of the intrinsic pathway. Apoptosis and the elimination of apoptotic cells are crucial factors in the maintenance of liver health. Apoptosis allows hepatocytes to die without provoking a potentially harmful inflammatory response. In contrast to necrosis, apoptosis is closely controlled and regulated via several mechanisms, including Fas/FasL interactions, the effects of cytokines such as TNF-α and TGF-β, and the influence of pro- and anti-apoptotic mitochondria-associated proteins of the B-cell lymphoma-2 (Bcl-2) family (8). The d-GalN/LPS challenge can significantly enhance the expression of Fas/FasL and TNF-α in the liver, suggesting that the hepatocyte apoptosis induced by d-GalN/LPS may be regulated by the death receptor pathway such as Fas/FasL interactions.

Caspases are broadly categorized into upstream regulatory caspases and downstream effector caspases. Caspase-3 is usually regarded as the downstream effector protease most important for the classic nuclear changes associated with apoptosis (14). The cleavage of the anti-apoptotic protein Bcl-2 into a proapoptotic form is a necessary step in Fas-mediated apoptosis, with caspase-3 mediating this processing (15). Caspase-3 is considered to be a key apoptotic ‘executioner’ enzyme in mammalian cells because its activation triggers the cascade of enzymatic events that culminates in the death of the cell (16). Fas-mediated cell death of hepatocytes is involved in many human liver pathologies, including hepatitis B virus-related cirrhosis, autoimmune hepatitis, acute liver failure, rejection of transplanted livers, and alcoholic and toxin-induced liver diseases. Moreover, caspase-3 is required for the initial events that occur in hepatocyte cell death following Fas ligation (17). In this study, the relative expression of Fas, FasL and caspase-3 proteins in murine liver were markedly elevated following d-GalN/LPS challenge. This finding suggests that the Fas-mediated death receptor pathway is one of the main mechanisms of hepatocyte apoptosis induced by d-GalN/LPS.

TGF-β is a multifunctional cytokine, whose numerous cell and tissue activities include cell-cycle control, differentiation, extracellular matrix formation, and the induction of apoptosis. The important role of TGF-β in orchestrating apoptosis in the liver is indicated by the hepatic fibrosis and apoptotic cell death of hepatocytes in transgenic mice that ectopically express TGF-β1 in the liver (18). TGF-β1-regulated apoptosis is cell-type and context-dependent, with TGF-β1 providing signals for cell survival or apoptosis. The molecular mechanisms underlying the role of TGF-β1 in apoptosis remain unclear. The proteins that primarily mediate the intracellular signaling of TGF-β1 are the members of the Smad family (19). Multiple apoptotic mediators and signaling pathways are involved in TGF-β-induced apoptosis. The activator protein (AP)-1 complex is also involved in TGF-β1 signaling for apoptosis (20). Bim is a crucial mediator of the apoptotic effects elicited by TGF-β (21). TGF-β1 signaling also cooperates with the death receptor apoptotic pathway (Fas and TNF). Moreover, the involvement of TGF-β1 in the production of oxidative stress and in preventing the inflammatory processes required for the clearance of apoptotic bodies is further evidence of its integration into apoptotic pathways (22). Since the expression of TGF-β1 in the d-GalN/LPS-induced apoptotic hepatocyte was significantly increased, it is suggested that the TGF-β signaling pathway plays a vital role in this process of the hepatocyte apoptosis induced by d-GalN/LPS.

There are several reasons that make a cell undergo apoptosis, including oxidative stress, DNA damage, accumulation of unfolded or misfolded proteins, occupation of death receptors by extracellular signals and lack of stimulation from other cells (12). Oxidative stress has been noted to contribute to the pathogenesis of acute hepatitis. Free radicals are toxic to hepatocytes and initiate a reactive oxygen species-mediated cascade causing hepatocyte cell death and leading to acute hepatitis (23,24). Oxygen-derived free radicals released from activated hepatic macrophages are the primary cause of d-GalN-induced liver damage (25,26). Increased production of reactive oxygen species has been reported in the primary culture of rat hepatocyte damage induced by d-GalN (27). d-GalN-induced hepatocyte injury is closely associated with the formation of oxidative stress. Oxidative stress is a factor that is capable of triggering apoptosis. Thus, the d-GalN/LPS-induced hepatocyte apoptosis is probably associated with oxidative stress.

This study has evaluated the d-GalN/LPS-induced hepatocyte apoptotic changes and clarified apoptosis-related gene expression (TNF-α, TGF-β1, Fas/FasL and caspase-3) in mice. The hepatocyte apoptosis induced by d-GalN/LPS is mainly regulated by the death receptor pathway. The TGF-β signaling pathway also plays a vital role in this process of hepatocyte apoptosis. These data provide a deeper understanding of the occurrence of the d-GalN/LPS-induced liver apoptosis.

## Figures and Tables

**Table I:** The serum ALT activity, TNF-α and TGF-β1 levels of hepatocyte apoptotic model induced by d-GalN/LPS in mice.

Groups	ALT (IU/l)	TNF-α (pg/ml)	TGF-β1 (pg/ml)
Vehicle control	15.91±1.13	18.65±4.92	20.82±4.49
d-GalN/LPS-treated	123.60±22.22^b^	134.83±80.35^a^	277.23±92.88^b^

Values are expressed as mean ± SD of seven replicates.

a P<0.01 and

b P<0.001 compared with the vehicle control.

SD, standard deviation; ALT, alanine aminotransferase; TNF-α, tumor necrosis factor-α; TGF-β1, transforming growth factor-β1; d-GalN, d-galactosamine; LPS, lipopolysaccharide.

**Table II:** The relative expression of Fas, FasL and caspase-3 proteins in liver tissues from the mice at 8 h after the d-GalN/LPS treatment.

Samples	Relative expression of Fas protein	Relative expression of FasL protein	Relative expression of caspase-3 protein
1	0.52±0.08	0.33±0.04	0.42±0.05
2	1.96±0.17	1.18±0.18	1.32±0.23
3	1.58±0.15	1.02±0.24	1.05±0.15
4	1.52±0.11	1.01±0.31	0.98±0.17
5	1.87±0.21	1.05±0.24	2.36±0.15
6	1.73±0.12	1.15±0.25	1.88±0.22
7	2.24±0.23	1.20±0.18	1.35±0.30
8	1.91±0.10	1.07±0.19	1.95±0.12

Data are expressed as mean ± SD. GAPDH antibody was used as an internal control. Sample 1 from the untreated control mice; Samples 2–8 from the mice at 8 h after the d-GalN/LPS injection. FasL, Fas ligand; d-GalN, galactosamine; LPS, lipopolysaccharide; SD, standard deviation; GAPDH, glyceraldehyde-3-phosphate dehydrogenase.
